# Short-term effect of air stacking and mechanical insufflation–exsufflation on lung function in patients with neuromuscular diseases

**DOI:** 10.1177/14799731221094619

**Published:** 2022-04-20

**Authors:** Esther S Veldhoen, Femke Vercoelen, Leandra Ros, Laura P Verweij-van den Oudenrijn, Roelie M Wösten-van Asperen, Erik HJ Hulzebos, Bart Bartels, Michael A Gaytant, Kors van der Ent, W Ludo van der Pol

**Affiliations:** 1 Pediatric Intensive Care Unit and Center of Home Mechanical Ventilation, Wilhelmina Children’s Hospital, |89098 University Medical Center Utrecht, Utrecht University, Utrecht, The Netherlands; 2 Department of Neurology, Brain Centre Rudolf Magnus, 89098 University Medical Center Utrecht, Utrecht University, Utrecht, The Netherlands; 3 Pediatric Intensive Care Unit, Wilhelmina Children’s Hospital, 89098 University Medical Center Utrecht, Utrecht, The Netherlands.; 4 Child Development and Exercise Center, Wilhelmina Children’s Hospital, 89098 University Medical Center, Utrecht University, Utrecht, The Netherlands; 5 Center of Home Mechanical Ventilation, Department of Pulmonology, 89098 University Medical Center Utrecht, Utrecht University, Utrecht, The Netherlands; 6 Department of Pediatric Pulmonology, Wilhelmina Children’s Hospital, 89098 University Medical Center, Utrecht University, Utrecht, The Netherlands

**Keywords:** Lung function, neuromuscular, airway clearance, home care

## Abstract

Air stacking (AS) and mechanical insufflation–exsufflation (MI-E) aim to increase cough efficacy by augmenting inspiratory lung volumes in patients with neuromuscular diseases (NMDs). We studied the short-term effect of AS and MI-E on lung function. We prospectively included NMD patients familiar with daily AS or MI-E use. Studied outcomes were forced vital capacity (FVC), forced expiratory volume in one second (FEV_1_), and peak expiratory flow (PEF) prior to, immediately after, and up to 2 h after treatment. Paired sample T-test and Wilcoxon signed-rank test was used. Sixty-seven patients participated. We observed increased FVC and FEV_1_ immediately after AS with a mean difference of respectively 0.090 L (95% CI 0.045; 0.135, *p* < .001) and 0.073 L (95% CI 0.017; 0.128, *p* = .012). Increased FVC immediately after MI-E (mean difference 0.059 L (95% CI 0.010; 0.109, *p* = .021) persisted 1 hour (mean difference 0.079 L (95% CI 0.034; 0.125, *p* = .003). The effect of treatment was more pronounced in patients diagnosed with Spinal Muscular Atrophy, compared to patients with Duchenne muscular dystrophy. AS and MI-E improved FVC immediately after treatment, which persisted 1 h after MI-E. There is insufficient evidence that short-lasting increases in FVC would explain the possible beneficial effect of AS and MI-E.

## Introduction

Respiratory muscle weakness causes cough impairment and respiratory failure and is a major cause of morbidity and mortality in patients with neuromuscular diseases (NMDs), including spinal muscular atrophy (SMA) and Duchenne muscular dystrophy (DMD).^1–4^ Cough impairment primarily compromises airway clearance and increases the risk of recurrent respiratory tract infections (RTIs) and hospital admissions.^1–6^ RTIs can further reduce lung function and this cycle may ultimately contribute to higher morbidity and mortality.^3,7^ The use of airway clearance techniques (ACTs) including air stacking (AS) and mechanical insufflation–exsufflation (MI-E) are therefore routinely used in patients with NMDs. Their use possibly results in a reduced number of RTIs and associated hospital admissions and shorter duration of hospital stays.^5,8–13^

International guidelines recommend initiation of ACTs when peak cough flow (PCF) falls below 270 L/min and/or forced vital capacity (FVC) is below 50% of predicted capacity,^2^ but do not specify preferred techniques. AS increases the inspiratory lung volume to its maximum by manually assisting the inspiration, resulting in an increased PCF.^14^ By increasing the inspiratory volume, AS enhances expiratory flow by a combination of static recoil and expiratory muscle recruitment.^2,15^ Advantages of AS include its low costs and availability. MI-E is often initiated when AS is impossible (e.g. in young children) or no longer effective.^3,11^ Unlike AS, MI-E also assists the expiration, by using a positive inspiratory pressure which is rapidly followed by a negative expiratory pressure. This rapid change in pressure mimics the flow changes that occur during a cough, thereby removing bronchial secretions.^3,16,17^ In comparison to AS, MI-E is much more expensive and not reimbursed in all countries.^9,18^ Studies show that both AS and MI-E improve cough strength immediately after treatment,^1,13,19–29^ yet the duration of this effect remains unclear.^24^ For this reason, we prospectively studied the effect of either AS and MI-E on lung function tests (LFTs) up to 2 h after ACT in patients with NMDs not naïve to this treatment. This hopefully helps to better understand the pathophysiological mechanism of these ACTs and may then improve and optimize ACT treatment in order to obtain maximal beneficial effect. We hypothesized that both AS and MI-E result in improved LFT, lasting at least 1 h after treatment.

## Methods

### Design and participants

In this prospective, single-center cohort study we included patients with NMDs without intercurrent RTI, who were already familiar with daily use of AS or MI-E at home at time of inclusion. All participants regularly attended the center for home mechanical ventilation at the University Medical Center Utrecht in the Netherlands, that serves large parts of the north-western, central and eastern parts of the Netherlands. Participants either used home mechanical ventilation or were at risk of chronic respiratory failure at the time of enrollment, that is, the second semester of 2020. Patients could not participate if they were unable to perform a spirometry, had a RTI at time of enrollment or when they did not understand Dutch or English, since this would interfere with informed consent. This study was approved by the institutional Medical Ethical Committee. Written informed consent was obtained from all participants and their parents in case of a minor.

### Airway clearance technique

All patients brought their own AS equipment and/or MI-E device. Patients used a self-inflating resuscitation bag (AMBU, Spur II, 1475 mL) for AS. The used MI-E device was the cough assist E70 (Philips Respironics). Patients were initiated on MI-E with greater exsufflation pressures than insufflation pressures and shorter insufflation time than exsufflation time. Settings were individualized based on patient comfort and improvement of cough strength.^18^ We instructed patients to perform ACT with the same number of sessions and repetitions as usual. In most cases, this consisted of three sets of five maximal inflation repetitions for AS and five cycles of five positive and negative pressures for MI-E. Settings of the MI-E were identical to settings at home as we wanted to study the real-life situation. Poor quality ACT maneuvers were excluded.

### Lung function tests

The primary outcome measures of this study were Forced Expiratory Volume in one second (FEV_1_), FVC, and peak expiratory flow (PEF), obtained with a handheld spirometer (CareFusion Microloop Spirometer) with an oronasal mask. Both absolute and standardized LFTs were measured and reported according to the European Respiratory Society Guidelines and the Global Lung Function Initiative.^30^ Although a recent Cochrane review suggested that Peak Cough Flow (PCF) improves after a range of cough augmenting techniques compared to unassisted cough,^31^ we decided not to use this as an outcome parameter. Measurement of PCF requires an additional maneuver which may result in fatigue with negative impact on LFT results. We used PEF as an alternative, because of the relation between PEF and PCF.^32^ Additionally, visual feedback on quality of LFT is possible with PEF measurement. We used tape-measured arm span as estimate for height in patients who were not able to stand without support.^33^ All subjects performed LFT in seated position, without corsets or braces. We performed spirometry before, immediately after, and one and 2 h after ACT. We documented the highest values out of three attempts.

### Statistical analysis

To describe baseline characteristics, we used descriptive statistics. We used IBM SPSS 25.0 and R (v3.6.0 with R Studio v1.2.1335). We used independent samples T-test for normally distributed variables and Mann-Whitney U test for parametric distributed variables to compare groups. All LFT parameters were tested for normality. We used paired sample T-test to determine improvement of LFT parameters after ACT for parameters with a normal distribution and Wilcoxon signed-rank test for parameters without normal distribution. Finally, we used linear regression to analyze the relationship between the effect of ACT and patient characteristics, such as the presence or severity of scoliosis and mechanical ventilation.

## Results

Sixty-nine patients were screened for eligibility. Two patients were excluded as we were not able to obtain reproducible LFT results, resulting in 67 included patients (54 children and 13 adults; median age 13.8 years (IQR 10.0; 17.2). The patient characteristics are shown in Table 1. Forty-eight patients (72%) used AS and 19 patients (28%) used MI-E. Patients in the MI-E group predominantly had SMA (89%), the remainder had Ullrich Congenital Muscular Dystrophy (UCMD) (11%). The AS group consisted of a more mixed group of NMDs, but the majority had SMA or DMD. We divided patients who used AS into three groups: SMA (type 1c and 2), DMD and other NMDs. Other NMDs included amyotrophic lateral sclerosis (*N* = 4), congenital myopathy (*N* = 4), limb girdle muscular dystrophy (*N* = 2), myotonic dystrophy (*N* = 2), hereditary motor sensory neuropathy (*N* = 2), and Kennedy’s disease (*N* = 1). Patients using MI-E were younger than patients treated with AS (median age 8.6 years (IQR 12.0; 18.3) and 15.3 years (IQR 12; 18.3), respectively (*p* < .001). Patients who used MI-E more frequently used mechanical ventilation (74%, compared to 46% for AS (*p* = .04). Scoliosis with a Cobb angle of more than 40° was more frequently present in the MI-E group (63%, compared to 37% for AS (*p* = .053). There were no significant differences in baseline standardized lung function parameters between the two groups.

**Table 1.** Baseline characteristics.

	AS (*N* = 48)			MI-E	(*N* = 19)
	SMA	DMD	Other	SMA	UCMD
	(*N* = 16)	(*N* = 14)	(*N* = 18)	(*N* = 17)	(*N* = 2)
Male gender, *N* (%)	8 (50)	14 (100)	7 (39)	13 (76)	2 (100)
Age in years, median (IQR)	12.4 (9.7; 14.9)	17.4 (15.1; 18.2)	29.6 (12.8; 55.8)	9.0 (5.5; 14.6)	8.2
Ventilation, *N* (%)					
No ventilation	8 (50)	5 (36)	13 (72)	3 (18)	2 (100)
Non-invasive	8 (50)	9 (64)	5 (28)	13 (76)	0 (0)
Invasive	0 (0)	0 (0)	0 (0)	1 (6)	0 (0)
Scoliosis, *N* (%)					
No scoliosis	0 (0)	5 (36)	12 (67)	0 (0)	0 (0)
Cobbs <40	5 (31)	6 (43)	1 (6)	5 (29)	2 (100)
Cobbs 40–80	9 (56)	2 (14)	4 (22)	11 (65)	0 (0)
Cobbs >80	2 (13)	0 (0)	0 (0)	1 (6)	0 (0)
Cobbs unknown	0 (0)	1 (7)	1 (6)	0 (0)	0 (0)
FEV_1_ in L, mean (SD)	0.861 (0.389)	1.160 (0.484)	1.15 (0.59)	0.542 (0.208)	0.410 (0.156)
FEV_1_ in %, median (IQR)	42 (27; 52)	32 (23; 45)	42 (32; 54)	35 (20; 49)	38
FVC in L, mean (SD)	0.978 (0.491)	1.47 (0.623)	1.39 (0.736)	0.651 (0.208)	0.510 (0.071)
FVC in %, median (IQR)	43 (24; 51)	38 (23; 45)	40 (32; 56)	38 (24; 46)	40
PEF in L/min, mean (SD)	102 (48)	114.7 (56.8)	122 (65.9)	102.3 (47.8)	64.5 (50.2)
PEF in %, median (IQR)	31 (23; 40)	20 (15; 33)	30 (22; 56)	25 (17; 38)	36

AS, Air stacking; DMD, Duchenne Muscular Dystrophy; FEV_1_, Forced Expiratory Volume in 1 s; FVC, Forced Vital Capacity; IQR, Inter Quartile Range; MI-E, Mechanical Insufflation–Exsufflation; Min, Minute; N, Number; PEF, Peak Expiratory Flow; SD, Standard Deviation; SMA, Spinal Muscular Atrophy; UCMD, Ullrich Congenital Muscular Dystrophy.

### Effects of air stacking

**Figure 1.**
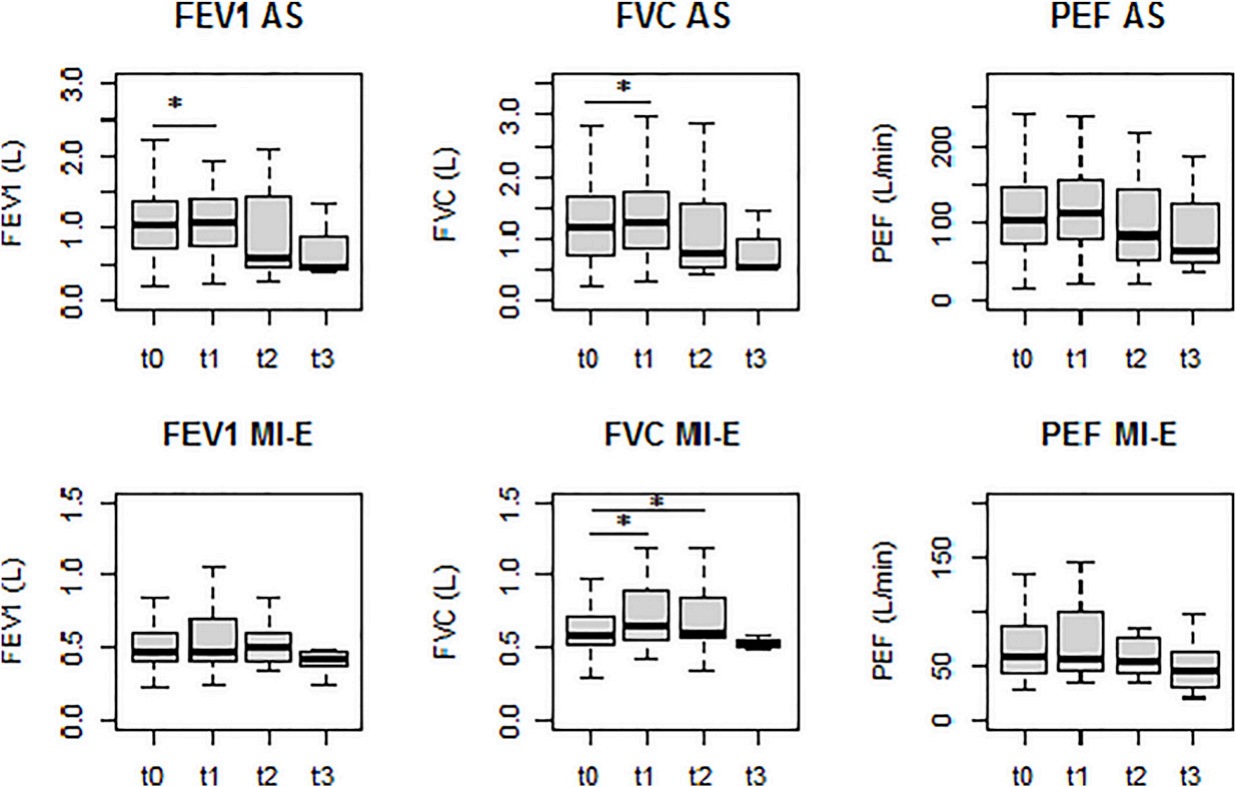
Effects of air stacking and mechanical insufflation-exsufflation on lung function immediately after (T1), 1 h after (T2) and 2 h (T3) after treatment compared to prior to air stacking or mechanical insufflation-exsufflation treatment (T0). AS, Air stacking; FEV_1_, Forced Expiratory Volume in 1 s; FVC, Forced Vital Capacity; MI-E, Mechanical Insufflation–Exsufflation; PEF, Peak Expiratory Flow; T0 = before AS or MI-E maneuver, T1 = immediately after AS or MI-E maneuver, T2 = 1 h after AS or MI-E maneuver, T3 = 2 h after AS or MI-E maneuver; * = statistically significant (*p* < .05).

We observed a significant improvement in FVC immediately after AS treatment with a mean difference of 0.090 L (95% CI 0.045; 0.135, *p* < .001). This effect was short-lasting, even 1 and 2 h after treatment FVC had returned to baseline levels. We observed a similar transient improvement of FEV_1_ immediately after AS with a mean difference of 0.073 L (95% CI 0.017; 0.128, *p* = .012) (Table 2, Figure 1), with return to baseline levels within hours. PEF was not different before and after AS treatment. The degree of scoliosis and use of mechanical ventilation did not influence outcome. Standardized LFT results before and after AS treatment are shown in Supplementary Table 1.

**Table 2.** Effects of air stacking and mechanical insufflation-exsufflation on lung function immediately after (T1), 1 h after (T2) and 2 h (T3) after treatment compared to prior to air stacking or mechanical insufflation-exsufflation treatment (T0).

	AS (*N* = 48)				MI-E (*N* = 19)			
	*N*	Mean diff	95% CI	*p*	*N*	Mean diff	95% CI	*p*
FEV_1_ (L)								
T0–T1	46	0.073	0.017; 0.128	.012*	19	0.028	−0.006; 0.063	.105
T0–T2	12	−0.020	−0.133; 0.093	.703	12	0.007	−0.058; 0.072	.825
T0–T3	3	−0.010	−0.204; 0.184	.845	8	0.026	−0.083; 0.135	.587
FVC (L)								
T0–T1	48	0.090	0.045; 0.135	.000*	18	0.059	0.010; 0.109	.021*
T0–T2	13	0.049	−0.053; 0.151	.313	12	0.079	0.034; 0.125	.003*
T0–T3	3	−0.070	−0.242; 0.102	.222	8	−0.008	−0.056; 0.041	.724
PEF (L/min)								
T0–T1	46	6.865	−4.423; 18.153	.227	18	−0.889	−9.639; 7.862	.833
T0–T2	12	−12.033	−38.852; 14.785	.345	12	−6.417	−25.411; 12.578	.473
T0–T3	4	3.333	−37.510; 44.177	.759	4	−2.625	−20.974; 15.724	.745

AS, Air stacking; CI, Confidence Interval; Diff, Difference; FEV_1_, Forced Expiratory Volume in 1 s, FVC, Forced Vital Capacity; MI-E, Mechanical Insufflation-Exsufflation; N, number; PEF, Peak Expiratory Flow; T0 = before AS or MI-E maneuver, T1 = immediately after AS or MI-E maneuver, T2 = 1 h after AS or MI-E maneuver, T3 = 2 h after AS or MI-E maneuver; * = statistically significant (*p* < .05).

### Effects of mechanical insufflation–exsufflation

Median pressures of MI-E used were +30 cmH_2_O (IQR 20; 39 cmH_2_O) and −35 cmH_2_O (IQR 30; 40). We observed a significant improvement of FVC immediately after treatment with a mean difference of 0.059 L (95% CI 0.010; 0.109, *p* = .021) (Table 2, Figure 1) that, in contrast to the AS group, persisted 1 h after treatment with a mean difference of 0.079 L (95% CI 0.034; 0.125, *p* = .003). All other LFT results, including FEV_1_, did not change after MI-E treatment. The degree of scoliosis and use of mechanical ventilation did not influence the effect of MI-E on lung function. Standardized LFT results before and after MI-E treatment are shown in Supplemental Table 1.

### Subgroup analyses: Duchenne muscular dystrophy and spinal muscular atrophy

We performed a subgroup analysis for disease categories. The 33 SMA patients constituted the largest patient group. Sixteen patients used AS and 17 used MI-E. In the AS group, all patients had SMA type 2, in the MI-E group five patients (29%) had SMA type 1c. SMA patients treated with MI-E, although not significant, were younger than SMA patients treated with AS (median age of 9.0 years (IQR 5.5; 14.6) and 12.4 years (IQR 9.7; 14.9), respectively (*p* = .201). The standardized baseline lung function parameters did not significantly differ between the AS and MI-E group (*p* > .2). FVC improved significantly immediately after AS and MI-E and remained so after 1 h in the MI-E group (Table 3).

**Table 3.** Subgroup analysis: Effect of air stacking and mechanical insufflation on lung function in patients with spinal muscular atrophy.

	AS (*N* = 16)				MI-E (*N* = 17)			
	*N*	Mean diff	95% CI	*p*	*N*	Mean diff	95% CI	*p*
FEV_1_ (L)								
T0–T1	14	0.786	0.010; 0.167	.077	17	0.031	−0.008; 0.070	.116
T0–T2	7	0.050	−0.046; 0.146	.250	11	0.020	−0.44; 0.084	.504
T0–T3	2	0.015	−0.811; 0.841	.856	8	0.263	−0.083; 0.135	.587
FVC (L)								
T0–T1	16	0.141	0.057; 0.226	.003*	16	0.056	0.001; 0.111	.046*
T0–T2	8	0.110	−0.027; 0.247	.099	11	0.087	0.041; 0.134	.002*
T0–T3	2	−0.050	−0.812; 0.712	.558	8	−0.008	−0.056; 0.041	.724
PEF (L/min)								
T0–T1	14	−5.571	−23.169; 12.026	.506	16	−2.250	−11.945; 7.445	.628
T0–T2	7	−4.286	−23.207; 14.636	.599	11	−1.000	−17.398; 15.398	.895
T0–T3	2	−6.000	−44.119; 32.119	.295	8	−2.625	−20.974; 15.723	.745

AS, Air stacking; CI, Confidence Interval; Diff, Difference; FEV_1_, Forced Expiratory Volume in 1 s; FVC, Forced Vital Capacity; MI-E, Mechanical Insufflation-Exsufflation; N, number; PEF, Peak Expiratory Flow; T0 = before AS or MI-E maneuver, T1 = immediately after AS or MI-E maneuver, T2 = 1 h after AS or MI-E maneuver, T3= 2 h after AS or MI-E maneuver; * = statistically significant (*p* < .05).

In contrast to the total and SMA group, we did not observe significant improvement in any of the LFT results performed by patients with DMD immediately after AS treatment (Table 4). We did not perform subgroup analysis in the mixed group, as the group was heterogeneous.

**Table 4.** Subgroup analysis: Effect of air stacking on lung function in patients with Duchenne muscular dystrophy.

	AS (*N* = 14)			
	*N*	Mean diff	95% CI	*p*
FEV_1_ (L)				
T0–T1	14	0.032	−0.125; 0.190	.666
T0–T2	2	0.060	−0.194; 0.314	.205
T0–T3				
FVC (L)				
T0–T1	14	0.061	−0.031; 0.153	.173
T0–T2	2	0.070	−0.692; 0.832	.451
T0–T3				
PEF (L/min)				
T0–T1	14	4.500	−12.534; 21.535	.578
T0–T2	2	27.600	−155.369; 210.569	.306

AS, Air stacking; CI, Confidence Interval; Diff, Difference; FEV_1_, Forced Expiratory Volume in 1 s; FVC, Forced Vital Capacity; N, number; PEF, Peak Expiratory Flow; T0 = before AS maneuver, T1 = immediately after AS maneuver, T2 = 1 h after AS maneuver, T3 = 2 h after AS maneuver.

## Discussion

### Summary of main findings

In this prospective cohort study, we observed that LFT improved immediately after AS (FVC and FEV_1_) and MI-E (FVC) and that this effect persisted for 1 h in the group that had used MI-E. Moreover, our results suggest that the effects of ACT may differ between NMDs, since the beneficial effects were most pronounced in SMA.

### (Dis)agreements with existing literature

Long-term effects of ACT have been previously, although not extensively, studied. Daily use of ACT probably helps to preserve vital capacity (VC) and reduces the annual decline in VC associated with NMDs including DMD and SMA.^9,34–36^ Importantly, ACT contributes to the prevention of RTIs and hospital admissions and may thus help to break the negative cycle of infections and declining LFT.^8,9^ Studies on immediate effect of AS^37^ and MI-E^38–41^ on VC showed conflicting results. In addition, some studies reported increased PCF, most likely secondary to increased VC, after AS and/or MI-E.^1,19–27,38–45^ To the best of our knowledge, only one study evaluated the VC up to 1 h after use of MI-E in nine patients with DMD. This study showed increased VC immediately after MI-E use, which returned to baseline within 1 h.^39^ We show that ACT improves LFT immediately, but that the duration of these effects may differ between techniques and disorders. This information is crucial for the design of future studies that may aim to develop tailored treatment strategies.

Our results showed more improvement in FVC in patients with SMA than with DMD, while the representation of other disorders was too limited to draw additional conclusions. This finding deserves further scrutiny in larger patient cohorts. NMDs differ not only in the degree, but also the pattern by which inspiratory and expiratory muscle groups are affected^46,47^ and what reserve capacity remains, which may be reflected by LFT. Other authors have suggested that cognitive and behavioral deficits may also influence efficacy of ACT and LFT outcomes, because active and conscient cooperation is necessary for optimal results.^48,49^ Cognitive defects are part of the DMD but not SMA phenotype and may therefore explain part of our findings. On the other hand, both poor quality ACT maneuvers and non-reproducible LFT results were excluded.

### Implications for future research and clinical practice

Our results demonstrate that FVC improved immediately after AS and MI-E, with even further improvement 1 h after MI-E treatment. Limited endurance for repeated muscle activities is a specific characteristic of SMA^50^ and may be a possible neuromuscular explanation for persistent improvement after 1 h, since the majority (89%) of the MI-E group had SMA. We do not think that the differences in baseline characteristics can explain the observed differences in the duration of ACT effects. First, baseline LFT parameters were comparable between the AS and MI-E groups. Second, the MI-E group contained the majority of patients with the most severe phenotype (i.e., SMA type 1c). To overcome the problem of fatigability in future studies, we would advise a 10–15 min break before performing LFTs after MI-E treatment. We can only speculate whether increased FVC of shorter duration could explain longer term effects of ACT. At this stage, there is insufficient evidence that short-lasting increases in FVC caused by the daily use of AS would explain the reduction RTIs. However, repeated AS and MI-E may help the preservation of lung and chest wall compliance that could be important in early phases of infections. Our study indicates similarities but also possible differences in efficacy of ACT that may be important for future clinical practice. The best instrument for future studies is probably a cross-over trial that would allow direct comparison of the effects of both ACTs in patients with SMA and possibly DMD. Multicenter collaborations would allow to study rarer neuromuscular disorders, including congenital muscular dystrophies and myopathies and limb girdle muscular dystrophies in more detail.

### Strengths and limitations

Strengths of this research include the prospective nature of the study. Patients were already familiar with the daily use AS or MI-E at home and with performing LFTing, thus excluding the possibility that increases of FVC reflect a learning effect. AS technique and MI-E settings used in our study were similar to the settings used at home, and thus reflect real-life situations. All patients were used to perform spirometry and all spirometry measurements were performed by the same professional, encouraging all patients in a similar way, thereby improving the quality of the data. This study is a relatively large study on a variety of rare NMDs and the study cohort was big enough to perform subgroup analysis. Finally, all study subjects were in good clinical condition at time of inclusion, therefore, the results were not influenced by RTI.

The present study has a few limitations. The COVID-19 pandemic negatively affected enrollment because fewer vulnerable patients attended the outpatient department. Despite the fact that none of the patients was naïve to spirometry, there might still be a learning effect in spirometry, which could alternatively explain part of our results. We think this is unlikely, because results showed an initial improvement immediately after treatment, followed by a return to baseline one and 2 h after treatment. Through the occurrence of fatigue, especially in SMA patients, effects of ACT may have been underestimated. Allowing patients to rest for a certain amount of time after ACT before spirometry may improve the results. Unfortunately, we suspected that the time required to stay in the hospital to perform this study would pose too much of a burden for some patients. Therefore, we were not able to measure the effect of ACT up to hours after treatment in all patients. This reduced power to demonstrate a positive effect after 1 and 2 h, especially in the AS group. Although the number of measurements after 2 h was small, we considered them important to report as available literature of measurements at this time interval is limited. Also, the subgroup with measurements after 2 h was more severely affected. Forced maneuvers during LFTing prior to ACT may have resulted in lung volume recruitment, thereby underestimating the effect of ACT on LFT results. Finally, the data is expressed as mean or median results for the patient cohort, obscuring individual variations. Our study population was heterogeneous, not only in type of NMD, but also in degree of lung restriction and possibly lung and chest wall compliance. Therefore, analyzing these variations in future studies might contribute to patient-tailored ACT and thereby optimize treatment for the individual patient. Despite these limitations, this study shows that a short-lasting improvement of FVC was observed after treatment with AS and MI-E in patients with NMDs.

## Conclusion

This prospective study demonstrated that AS and MI-E improves FVC immediately after treatment. This effect persisted 1 h after MI-E treatment. Additionally, the effect of ACT was more pronounced in patients diagnosed with SMA, compared to patients diagnosed with DMD. At this stage, there is insufficient evidence that short-lasting increases in FVC caused by the daily use of AS or MI-E would explain the possible beneficial effect.

## Supplemental Material

Supplemental Material - Short-term effect of air stacking and mechanical insufflation–exsufflation on lung function in patients with neuromuscular diseases Click here for additional data file. Supplementary Material for Short term effect of air stacking and mechanical insufflation–exsufflation on lung function in patients with neuromuscular diseases by Esther S Veldhoen, Femke Vercoelen, Leandra Ros, Laura P Verweij-van den Oudenrijn, Roelie M Wösten-van Asperen, Erik HJ Hulzebos, Bart Bartels, Michael A Gaytant, Kors van der Ent, and W Ludo van der Pol in Chronic Respiratory Disease.
