# Supplementary material for: Short-term effect of air stacking and mechanical insufflation–exsufflation on lung function in patients with neuromuscular diseases
Source: Chron Respir Dis. 2022 Apr 20;19:14799731221094619. doi: 10.1177/14799731221094619 (PMC9024083; doi:10.1177/14799731221094619)
Supplement: Supplemental Material - Short-term effect of air stacking and mechanical insufflation–exsufflation on lung function in patients with neuromuscular diseases [file sj-pdf-1-crd-10.1177_14799731221094619.pdf]

Supplementary table 1: Effects of air stacking and mechanical insufflation-exsufflation on standardized lung function results immediately after (T1), one hour after (T2) and two hours (T3) after treatment compared to prior to air stacking or mechanical insufflation-exsufflation treatment (T0)

	**AS** (N=48)			**MI-E** (N=19)		
	N	Median (IQR)	P (Z)	N	Median (IQR)	P (Z)
FEV_1 (%)						
T0	47	38 (27; 51)	NA	19	35 (22; 49)	NA
T1	47	40 (28; 55)	0.005* (-2.7821	19	36 (24; 46)	0.257 (-1.134)
T2	13	30 (21; 49)	0.875 (-0.157)	12	36 (27; 45)	0.838 (-0.204)
T3	3	37	1.000 (0.000)	8	33 (21; 38)	0.944 (-0.070)
FVC (%)						
T0	48	40 (27; 51)	NA	19	38 (28; 45)	NA
T1	48	44 (30; 51)	0.000* (-3.851)	18	40 (31; 50)	0.014* (-2.456)
T2	13	31 (24; 40)	0.349 (-0.936)	12	43 (30; 52)	0.005* (-2.803)
T3	3	39	0.285 (-1.069)	8	36 (23; 40)	0.833 (-0.211)
PEF (%)						
T0	47	29 (18; 38)	NA	19	25 (16; 40)	NA
T1	47	30 (20; 39)	0.211 (-1.251)	18	26 (18; 37)	0.758 (-0.308)
T2	12	32 (14; 34)	0.326 (0.982)	12	26 (19; 30)	0.906 (-0.118)
T3	3	30	0.593 (-0.535)	8	26 (19; 30)	0.395 (-0.851)

Legend: *AS= Air stacking, FEV_1= Forced Expiratory Volume in 1 second, FVC= Forced Vital Capacity, IQR= Interquartile range, MI-E= Mechanical Insufflation-Exsufflation, N= number, NA= Not applicable, PEF= Peak Expiratory Flow, T0= before AS or MI-E maneuver, T1= immediately after AS or MI-E maneuver, T2= 1 hour after AS or MI-E maneuver, T3= 2 hours after AS or MI-E maneuver, *= statistically significant (P<0.05)*

Supplementary table 2 Subgroup analysis: Effect of air stacking and mechanical insufflation on standardized lung function in patients with Spinal Muscular Atrophy

	**AS** (N=16)			**MI-E** (N=17)		
	N	Median (IQR)	P (Z)	N	Median (IQR)	P (Z)
FEV_1 (%)						
T0	15	42 (27; 52)	NA	17	35 (20; 49)	NA
T1	15	43 (28; 52)	0.135 (-1.493)	17	36 (23; 44)	0.295 (-1.047)
T2	8	40 (18; 49)	0.309 (-1.018)	11	36 (26; 45)	0.440 (-0.771)
T3	2	40	0.655 (-0.447)	8	33 (21; 38)	0.944 (-0.070)
FVC (%)						
T0	16	43 (24; 51)	NA	17	38 (24; 46)	NA

	N	Median (IQR)	p (Z)	N	Median (IQR)	p (Z)
T1	16	46 (30; 50)	0.007* (-2.692)	16	40 (30; 50)	0.039* (-2.063)
T2	8	43 (23; 51)	0.128 (-1.521)	11	43 (29; 53)	0.005* (-2.805)
T3	2	41	0.655 (-0.447)	8	36 (23; 40)	0.833 (-0.211)
PEF (%)						
T0	15	33 (25; 43)	NA	17	25 (17; 36)	NA
T1	15	33 (15; 39)	0.638 (-0.471)	16	25 (17; 36)	0.864 (-0.171)
T2	8	31 (11; 34)	0.498 (-0.677)	11	26 (19; 31)	0.688 (-0.401)
T3	2	38	0.655 (-0.447)	8	19 (13; 28)	0.395 (-0.851)

Legend: *AS= Air stacking, FEV_1= Forced Expiratory Volume in 1 second, FVC= Forced Vital Capacity, IQR= Interquartile range, MI-E= Mechanical Insufflation-Exsufflation, N= number, NA= not applicable, PEF= Peak Expiratory Flow, T0= before AS or MI-E maneuver, T1= immediately after AS or MI-E maneuver, T2= 1 hour after AS or MI-E maneuver, T3= 2 hours after AS or MI-E maneuver, *= statistically significant (P<0.05)*

Supplementary table 3 Subgroup analysis: Effect of air stacking on standardized lung function in patients with Duchenne Muscular Dystrophy

		**DMD** (N=14)	
	N	Median (IQR)	p (Z)
FEV_1 (%)			
T0	14	32 (23; 45)	NA
T1	14	34 (21; 48)	0.699 (-0.387)
T2	2	21	0.157 (-1.414)
T3	1		
FVC (%)			
T0	14	38 (23; 45)	NA
T1	14	39 (22; 48)	0.124 (-1.539)
T2	2	20	0.317 (-1.000)
T3	1		
PEF (%)			
T0	14	20 (15; 33)	NA
T1	14	25 (15; 30)	0.552 (-0.595)
T2	2	22	0.180 (-1.342)
T3	1		

Legend: *AS= Air stacking,* FEV_1*= Forced Expiratory Volume in 1 second, FVC= Forced Vital Capacity, IQR= Interquartile range, MI-E= Mechanical Insufflation-Exsufflation, N= number, NA= Not applicable, PEF= Peak Expiratory Flow, T0= before AS or MI-E maneuver, T1= immediately after AS or MI-E maneuver, T2= 1 hour after AS or MI-E maneuver, T3= 2 hours after AS or MI-E maneuver*
